# From Malaise to Oral Pain: A Case Report on Oral Mucosal Melanoma

**DOI:** 10.1155/crid/4381566

**Published:** 2026-10-03

**Authors:** Maya Ramachandran, Joy Chang, Pratibha Shukla, Sarah A. Weiss, Mahnaz Fatahzadeh

**Affiliations:** ^1^ Department of Dental Robert Wood Johnson University Hospital New Brunswick New Jersey USA rwjuh.edu; ^2^ Department of Pathology Rutgers New Jersey Medical School and University Hospital Newark Newark New Jersey USA; ^3^ Department of Medical Oncology Rutgers Cancer Institute New Brunswick New Jersey USA; ^4^ Department of Diagnostic Sciences Rutgers School of Dental Medicine Newark New Jersey USA rutgers.edu

**Keywords:** liver metastasis, oral cancer oral mucosal melanoma, oral melanoma

## Abstract

**Background:**

Oral melanoma is a rare form of melanoma that typically presents as a black‐brown macule or nodule with variable pigmentation and occasional depigmented areas. Oral melanoma typically affects the maxillary gingiva, palate, and retromolar trigone, and tends to be aggressive with high propensity for distant spread and poor prognosis. We report a patient with metastatic visceral melanoma who, upon presentation for dental care, was found to also have advanced gingival melanoma with bone involvement. This may support the oral cavity as the primary site and represents a missed opportunity for early diagnosis.

**Case Description:**

A 61‐year‐old male presented to the emergency department with 20 pound weight loss and groin pain. Imaging work‐up demonstrated liver masses, retroperitoneal lymphadenopathy, and lung nodules suspicious for metastatic disease. He returned to the emergency department 3 weeks later with a toothache and was instructed to follow up in the dental clinic in the hospital for tooth extractions. At this visit, discoloration of the gingiva was noted in the anterior maxilla (Teeth #6–9), and a biopsy confirmed the likely primary malignant site: oral melanoma.

**Practical Implications:**

This case highlights the importance of detecting melanoma in the oral cavity. Although rare, oral melanoma has a low five‐year survival rate, and dentists must conduct a thorough head and neck exam with proper referral and follow up for any suspicious lesions.

## 1. Introduction

Oral melanoma is a rare form of melanoma, accounting for 0.2%–0.8% of all melanomas [1, 2] and 1%–2% of all oral malignancies [1, 3]. Unlike cutaneous melanoma, it often lacks early visible signs and can mimic benign pigmented lesions or other intraoral conditions, making timely diagnosis particularly challenging. The 5‐year survival rate remains low, ranging from 15% to 38% [4], largely due to delayed detection and early metastasis.

Oral mucosal melanoma (OMM) most commonly affects older men, with a mean age of approximately 60 years, although cases have been reported across a wide age range, including adolescents and the elderly [5, 6]. Oral melanoma is more common in non‐Hispanic White individuals.

Due to the neural crest origin of melanocytes, melanomas may arise in noncutaneous locations containing neural crest derivatives, including the central nervous system and gastrointestinal tract [7]. Unlike cutaneous melanoma, oral melanoma is not associated with ultraviolet (UV) radiation exposure. Its pathogenesis is thought to involve malignant transformation of melanocytes in the basal layer of the oral epithelium, influenced by genetic and epigenetic factors [8].

These tumors typically arise on the hard palate or maxillary gingiva and may be asymptomatic in early stages. By the time they are clinically recognized—often due to swelling, bleeding, or tooth mobility—they may have already invaded local tissues or metastasized [9]. Common metastatic sites include the regional lymph nodes, lungs, and liver [7].

This case is particularly notable because metastatic visceral melanoma was identified prior to recognition of the oral primary lesion, underscoring the diagnostic challenges of OMM and the critical role of dental evaluation in patients with melanoma of unknown primary.

## 2. Clinical Case

A 61‐year‐old Bengali male presented to the emergency department with groin pain. His past medical history included hypertension, Type 2 diabetes mellitus, hypothyroidism, and gastroesophageal reflux disease (GERD). His medications included amlodipine, metformin, glipizide, nicotine polacrilex, and omeprazole. He had no known drug allergies.

The patient was a former smoker with a 40 pack‐year history (1 pack per day for 40 years), having quit 1 year prior and was currently using nicotine replacement therapy. On presentation, it was noted that he had unintentionally lost 20 pounds over the past year.

Although the groin pain was ultimately determined to be insignificant, CT imaging revealed masses in the liver. Medical oncology was consulted for further evaluation. Additional imaging revealed retroperitoneal lymphadenopathy, pulmonary masses, and trace pelvic ascites. Given the concern for metastatic disease, the patient was referred for outpatient biopsy and oncology follow‐up.

Biopsy of a liver metastasis confirmed metastatic melanoma, and the patient was subsequently started on combination immunotherapy with ipilimumab and nivolumab. Further imaging showed metastatic involvement of the lungs, kidneys, spleen, splenic hilum, rectus muscle, and chest wall. At this stage, the primary site of melanoma remained unclear, as no cutaneous lesions were identified. Three weeks later, the patient returned to the hospital with complaints of oral pain. Examination in the emergency department revealed a dark gingival pigmentation in the region of Teeth #6–9, and he was referred to the dental clinic for evaluation and extraction of nonrestorable teeth.

On presentation to dental clinic, the patient appeared frail and complained of fatigue, poor appetite, weight loss, and progressive loosening of his upper front teeth. He reported only receiving sporadic dental care in the past and had noticed the change in the color of his gingiva months prior to his dental visit. Intraoral exam revealed black discoloration of upper labial vestibule and incisor teeth gingiva as well as an exophytic mass of similar color palatal to Teeth #7–9 (Figure 1). The affected teeth had Grade 3 mobility with no or minimal bone support on imaging (Figures 2 and 3).

**Figure 1:**
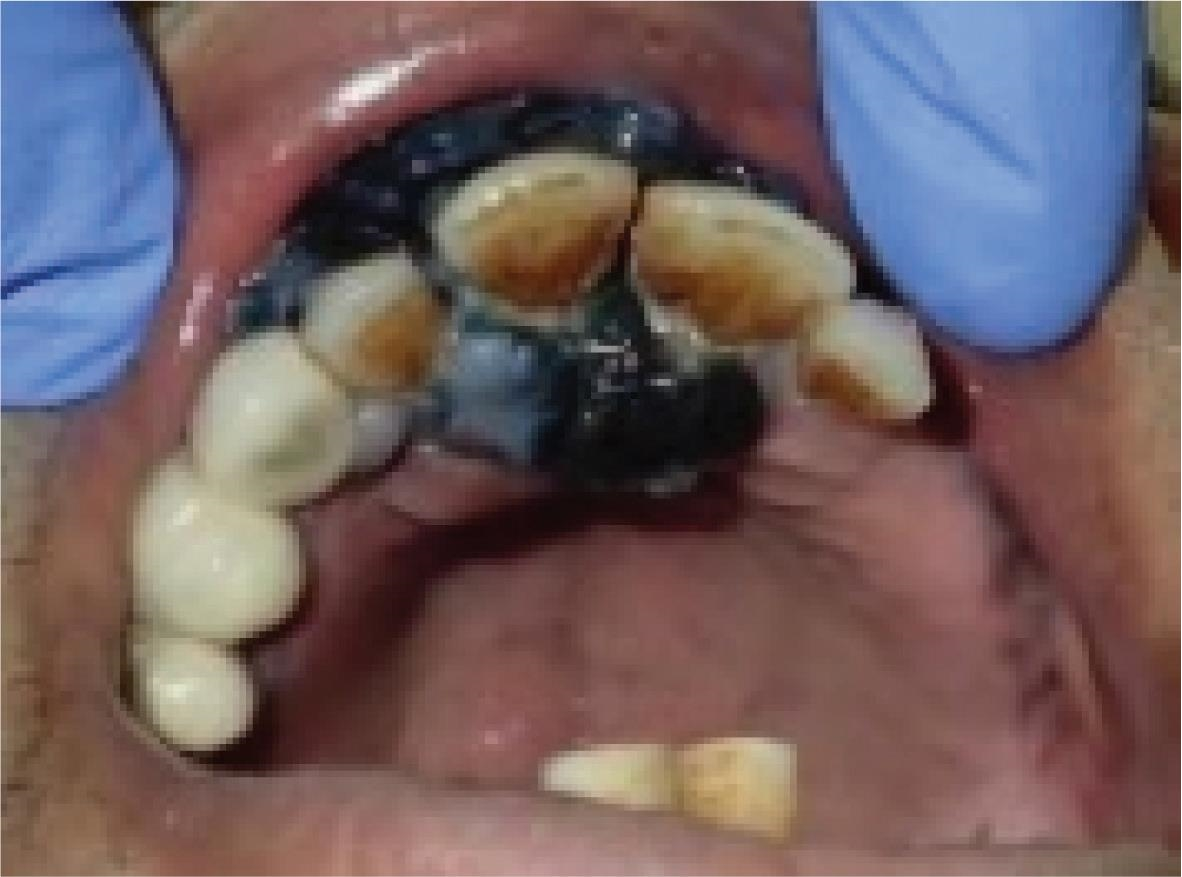
Clinical photo illustrating black discoloration of maxillary labial vestibule and a heavily pigmented exophytic mass palatal to upper front teeth at the initial presentation of the patient to dental clinic.

**Figure 2:**
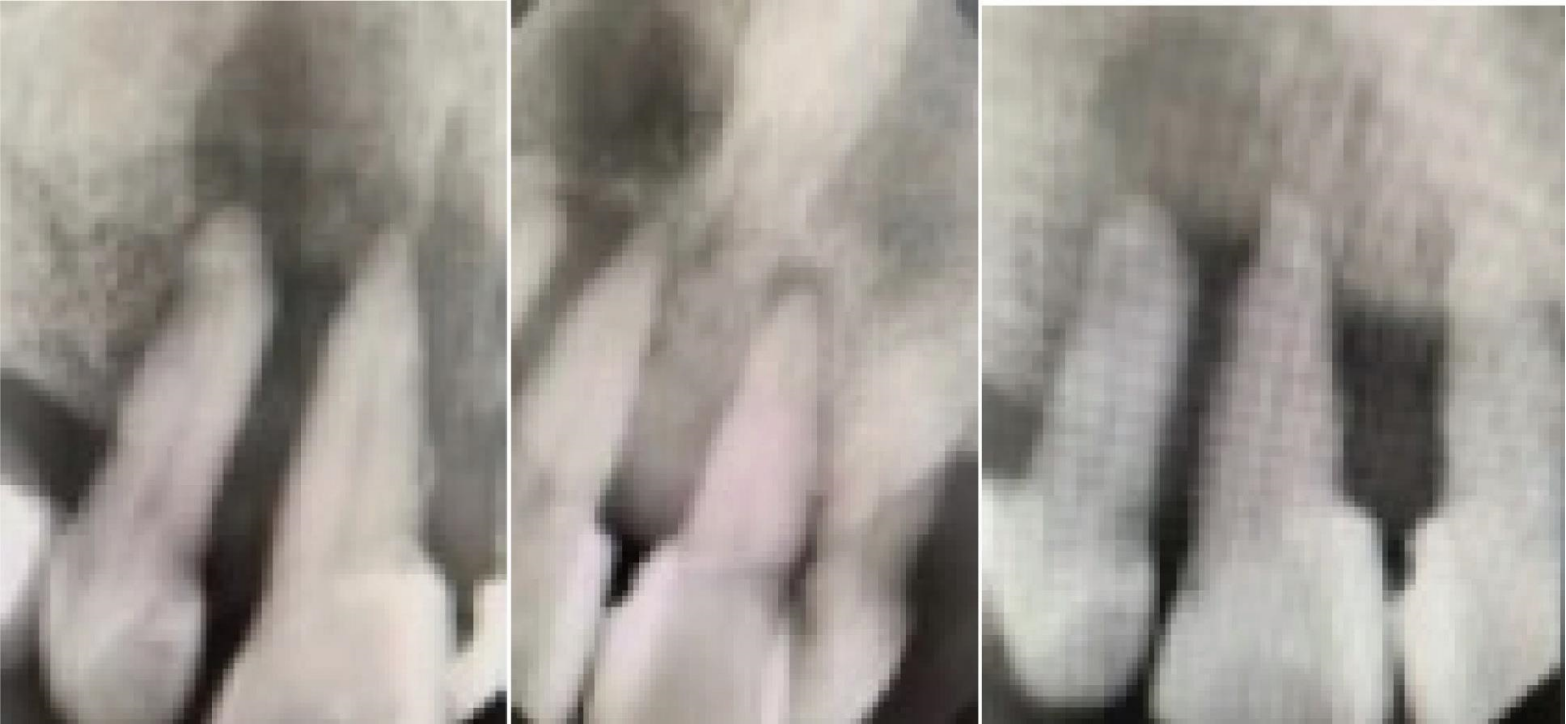
Radiographic image of the highly mobile maxillary anterior teeth with severe bone loss.

**Figure 3:**
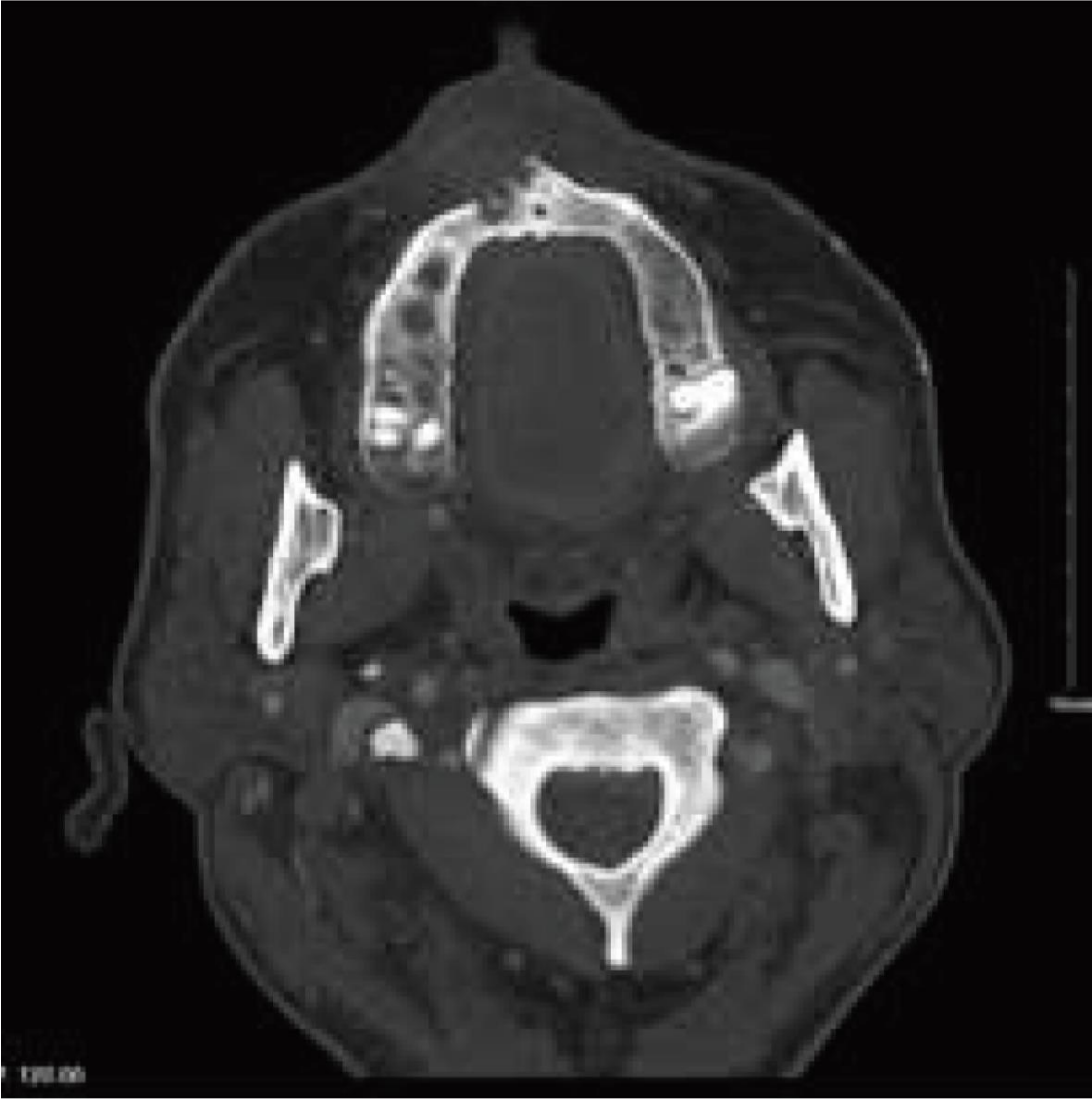
Axial view computed tomography imaging of facial bones disclosing a soft tissue mass along the upper gingiva/labial mucosa centered slightly to the right of midline which is highlighted by ∗. This measures at least 1.0 × 3.0 cm axially and approximately 2 cm craniocaudally.

An incisional biopsy of the maxillary anterior gingiva and vestibule was subsequently performed. Histopathological analysis revealed malignant melanoma. Gingival mucosa was infiltrated by sheets of heavily pigmented cells with atypical nuclei and numerous mitotic figures (Figure 4a). Lesional cells stained diffusely positive for SOX‐10, HMB‐45, and Melan‐A (Figure 4b–d) and showed a high proliferative index with Ki‐67 staining, consistent with malignant melanoma. A red chromogen was utilized for antigen detection instead of routine brown chromogen to distinguish the target antigen staining from brown melanin pigment present in the tumor cells.

**Figure 4:**
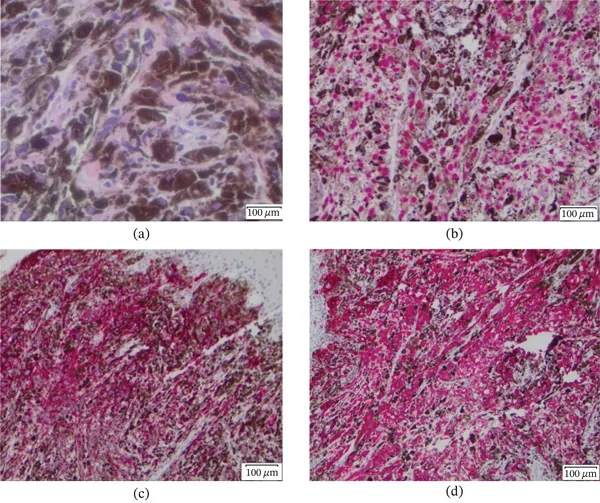
(a) Pigmented cells have enlarged, pleomorphic nuclei with prominent nucleoli. Frequent mitotic figures are encountered. (b) Lesional cells stain positive with SOX10 (nuclear staining with red chromogen). (c) Lesional cells stain positive for HMB45 (cytoplasmic staining and red chromogen). (d) Lesional cells stain positive with Melan‐A (cytoplasmic staining and red chromogen).

Following three cycles of ipilimumab and nivolumab, imaging demonstrated disease progression. The patient′s treatment was subsequently changed to pembrolizumab and lenvatinib. Nearly a year later, the patient was admitted to the hospital once again, this time due to poor appetite. The dental team was consulted to evaluate a lesion on the hard palate. The patient reported intermittent pain localized to the left side of the hard palate, particularly during eating, and experienced tenderness upon palpation of the lesion.

Extraoral examination revealed bilateral, fixed, firm nodules approximately 1 cm in diameter at the angles of the mandible (Figure 5). Follow up computed tomography of the facial bones disclosed multiple pathologic lymph nodes in the right submandibular region and right posterior triangle (Figure 6). Intraorally, edentulous alveolar mucosa corresponding to Tooth #9 appeared blue‐black and a 3 × 1 cm growth with rolled borders and central perforation was evident on the left hard palate (Figure 7) likely representing another site of disease spread.

**Figure 5:**
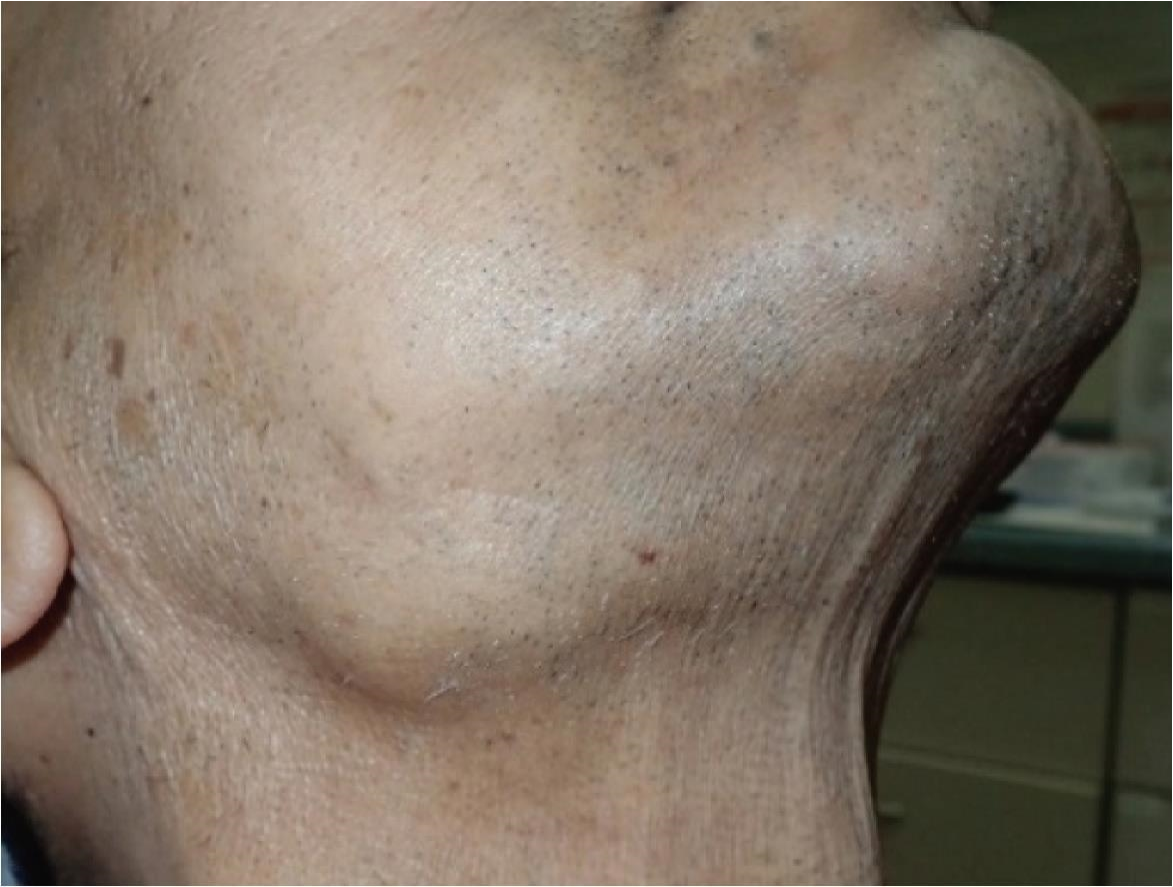
Clinical photo illustrating a fixed, firm, nonmoveable submucosal swelling of about 1.5 × 1 cm on the right angle of the mandible.

**Figure 6:**
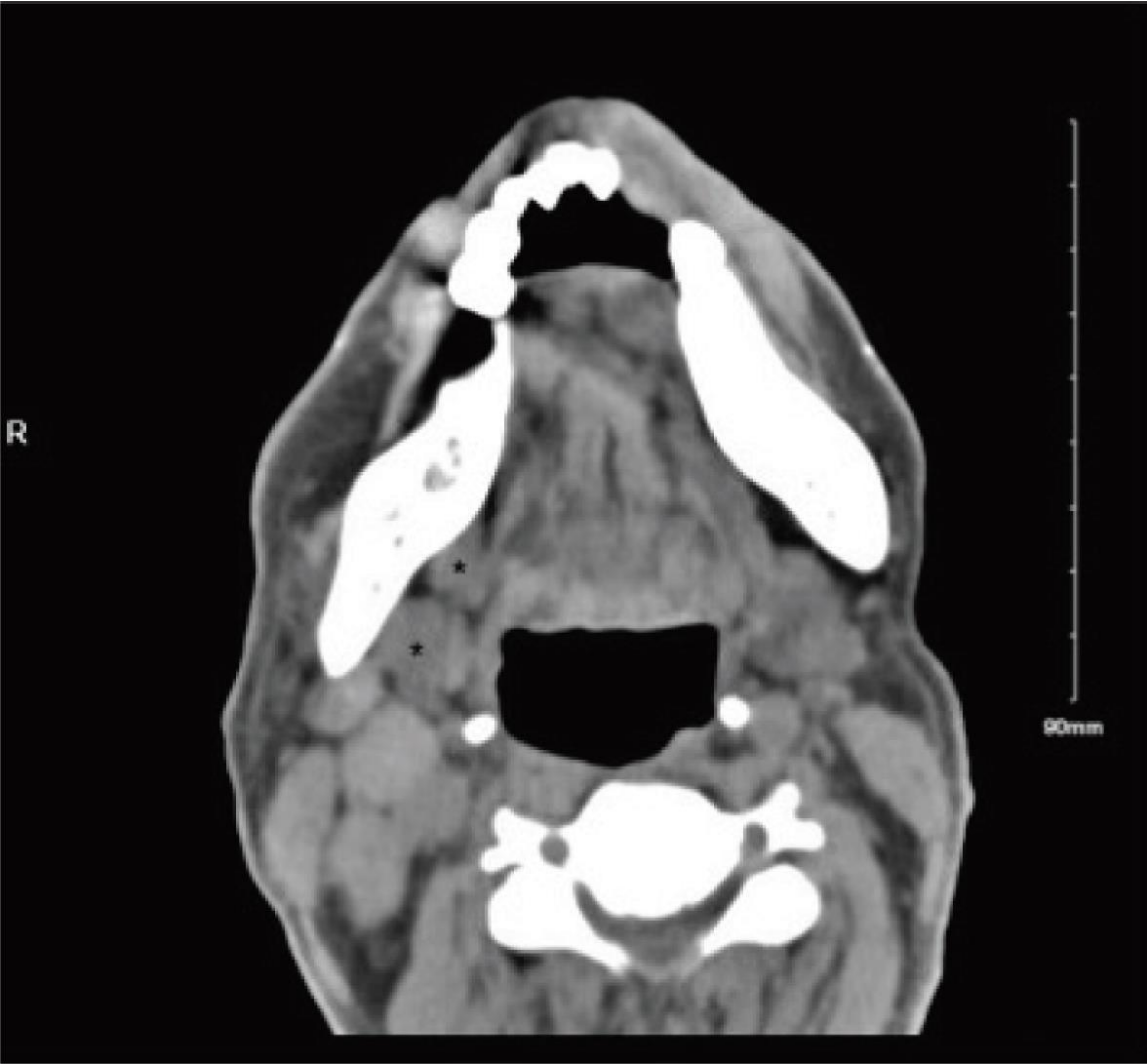
Axial view computed tomography of facial bones disclosing enlarged right submandibular lymph nodes annotated with an ∗.

**Figure 7:**
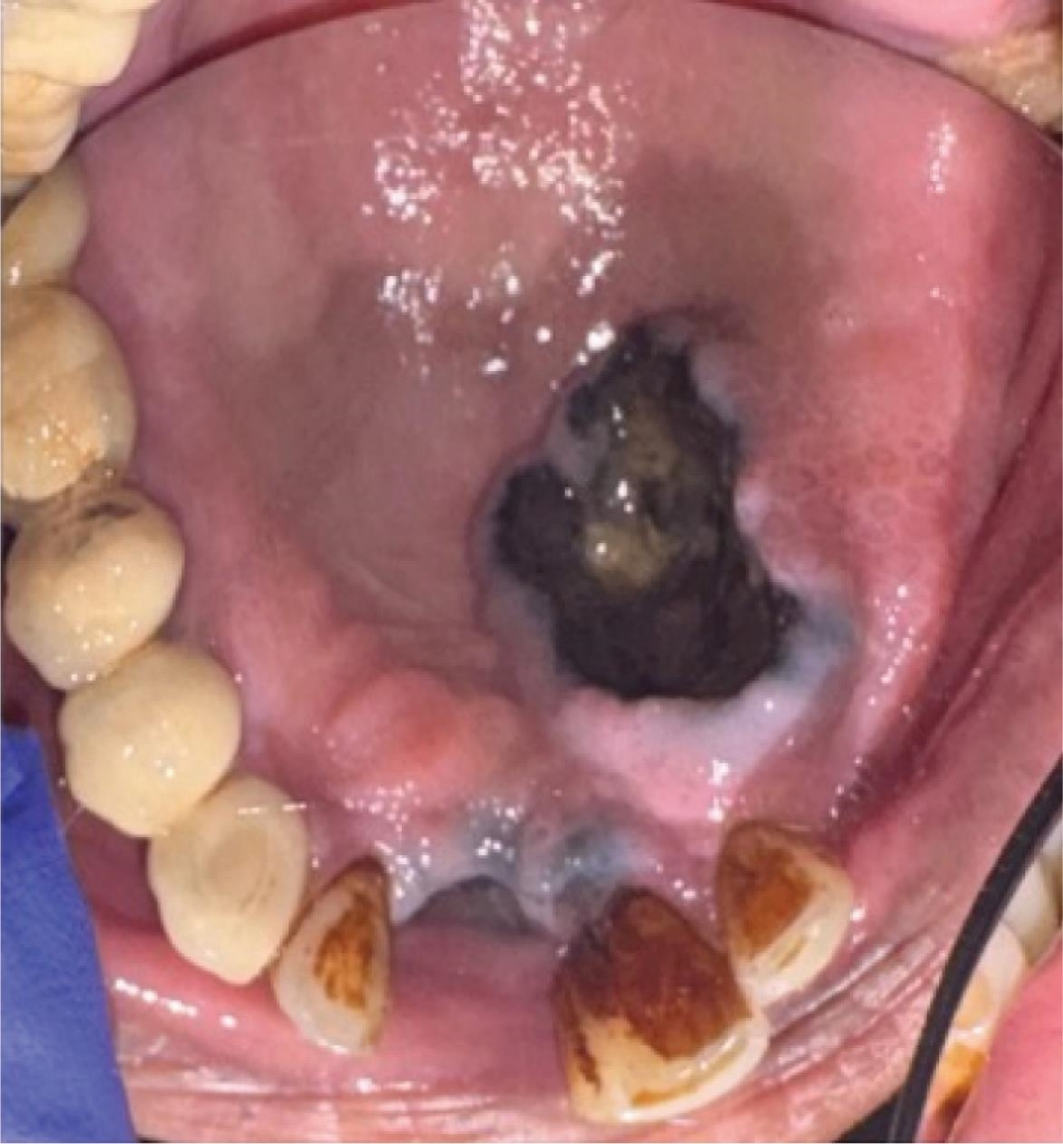
Clinical photo illustrating a 3 × 1 cm pigmented mass on the left hard palate with rolled borders and evidence of underlying bone perforation.

The patient reported limited oral intake and continued to experience progressive weight loss and physical decline. A PET CT was ordered and demonstrated ongoing progression of metastatic disease involving the chest, abdomen, and pelvis. He was referred to palliative care for pain management and was prescribed oxycodone 2.5–5 mg every 4 h, 2% viscous lidocaine, and acetaminophen, all as needed.

The patient elected to enroll in Phase I clinical trial of immune‐based therapy for patients with advanced or metastatic solid tumors but unfortunately passed away.

The chronology of the patient′s presentation, diagnostic workup, treatment, disease progression, and outcome is summarized in Table 1.

**Table 1:** Timeline of clinical presentation, diagnosis, treatment, and outcome.

Approximate time point	Clinical event	Key findings/management
Initial ED presentation	Patient presented with groin pain and constitutional symptoms	Reported unintentional 20‐lb weight loss over the prior year. CT imaging revealed liver masses suspicious for metastatic disease.
Initial oncologic workup	Further imaging performed	Retroperitoneal lymphadenopathy, pulmonary nodules/masses, and trace pelvic ascites were identified.
Shortly after initial workup	Liver biopsy	Biopsy confirmed metastatic melanoma. No cutaneous primary lesion was identified at that time.
After diagnosis of metastatic melanoma	Systemic therapy initiated	Combination immunotherapy with ipilimumab and nivolumab was started.
Approximately 3 weeks after initial ED visit	Return to hospital with oral pain/toothache	Dark gingival pigmentation was noted in the anterior maxilla, involving the region of Teeth #6–9. Patient was referred for dental evaluation.
Dental clinic evaluation	Oral examination and imaging	Blue‐black pigmentation of the maxillary anterior gingiva/labial vestibule and palatal exophytic mass were noted. Teeth #7–9 showed severe mobility with minimal bone support.
Following dental evaluation	Oral incisional biopsy	Biopsy of the maxillary anterior gingiva/vestibule confirmed malignant melanoma with SOX‐10, HMB‐45, and Melan‐A positivity and high Ki‐67 proliferative index.
After three cycles of ipilimumab/nivolumab	Restaging imaging	Imaging demonstrated progression of metastatic disease.
Subsequent treatment	Change in systemic therapy	Therapy was changed to pembrolizumab and lenvatinib.
Nearly 1 year later	Hospital admission for poor appetite and oral pain	New hard palate lesion was identified; extraoral exam showed bilateral firm nodules near the mandibular angles. CT showed pathologic cervical/submandibular lymph nodes.
Later disease course	PET/CT and palliative referral	PET/CT showed continued progression involving the chest, abdomen, and pelvis. Patient was referred to palliative care for symptom management.
Final course	Clinical trial enrollment and death	Patient elected enrollment in a phase I immune‐based therapy trial for advanced/metastatic solid tumors but subsequently passed away.

## 3. Discussion

Melanoma is a malignant neoplasm arising from melanocytes, most commonly affecting sun‐exposed areas of the skin. However, melanocytes are also present in mucosal tissues, including the oral cavity.

Non‐Hispanic White individuals have the highest absolute incidence rates of oral melanoma, primarily due to their higher overall melanoma rates. However, Asian/Pacific Islander, Black, and Hispanic populations have a higher proportion of mucosal melanomas—including oral melanoma—relative to their total melanoma cases [10]. A study by Altieri et al. [10] reported that mucosal melanomas constitute approximately 15% of all melanomas in Asian/Pacific Islanders, 9% in non‐Hispanic Blacks, and 4% in Hispanics, compared to only about 1% in non‐Hispanic Whites.

Importantly, increased melanin levels—which offer protection against UV radiation—do not influence the risk of developing oral melanoma [9]. This is because oral melanoma arises in mucosal tissues that are not exposed to UV light [9]. Although benign intraoral melanocytic proliferations (nevi) do occur and may serve as precursors in rare cases, the progression from nevus to melanoma in the oral cavity remains poorly understood. Currently, most oral melanomas are believed to arise de novo, without an identifiable precursor lesion [9]. In rare instances, malignant transformation involves the clonal expansion of melanocytes—either within an existing nevus or among isolated basal layer melanocytes—that acquire a selective growth advantage and begin uncontrolled proliferation [9]. The etiology of oral melanoma remains largely unknown, and the mechanisms underlying malignant transformation in mucosal melanocytes continue to be an area of active research.

Clinically, oral melanoma often presents as an asymptomatic macule with variable pigmentation. Classic features include a dark brown to black lesion, frequently involving the maxillary gingiva or hard palate. These lesions typically have irregular and uneven borders and may exhibit loss of pigmentation, ulceration, or bleeding. In some cases, approximately 37%, OMM arises from a pre‐existing pigmented lesion [11]. Differential diagnoses for isolated pigmented lesions include benign entities such as amalgam tattoo, melanotic macule, melanocytic nevus, and melanoacanthoma. After a thorough clinical history taking to determine if this lesion is isolated or part of a systemic condition, dentists should evaluate the size, color, border, and induration of these lesions. Lesions that are isolated with irregular borders and color variation should be referred for biopsy. The American Academy of Oral and Maxillofacial Pathology recommends biopsy of any persistent, solitary pigmented lesion—particularly in high‐risk sites such as the palate and gingiva [12, 13].

As OMM progresses, the lesion may become nodular and exhibit invasive characteristics, including erosion of underlying bone [14]. In the present case, the patient exhibited diffuse blue‐black pigmentation in the anterior maxilla (Teeth #6–9), along with a 3 × 1 cm endophytic lesion with rolled borders and associated bone perforation.

Determining whether the oral lesion represented a primary OMM or an oral metastasis from an unknown primary site was challenging. Diagnostic criteria proposed for primary oral melanoma include evidence of malignant melanoma in the oral mucosa, intraepithelial or junctional activity when present, and exclusion of another primary melanoma site [14]. In the present case, malignant melanoma was confirmed within the oral mucosa; however, significant junctional activity or an unequivocal melanoma in situ component was not identified histologically; therefore, clinicopathologic correlation and exclusion of another primary site are essential. The oral cavity cannot be designated as the definitive primary site with absolute certainty, and the possibility of metastatic melanoma to the oral cavity or melanoma of unknown primary cannot be completely excluded.

Nevertheless, several clinicopathologic features support the oral cavity as the presumptive primary site. First, no cutaneous, ocular, or other mucosal primary lesion was identified despite clinical and radiographic evaluation. Second, the patient reported noticing gingival discoloration months before the dental visit, suggesting that the oral lesion may have been present prior to it being formally evaluated and biopsied. Third, the lesion involved a common site for OMM, namely the maxillary gingiva/anterior palate, and demonstrated locally destructive behavior with severe alveolar bone loss and marked tooth mobility. Finally, OMMs are often diagnosed late and may present after regional or distant metastasis has already occurred. For these reasons, the oral lesion was interpreted as the most likely, though not definitively proven, primary site.

Immunohistochemical diagnosis of OMM can be challenging, as no single marker reliably detects all cases. Commonly used markers include S‐100, Melan‐A, MITF, tyrosinase, and HMB‐45, each showing variable expression [15]. SOX‐10 has emerged as a useful marker due to its high sensitivity in OMM, though it has moderate specificity in broader mucosal melanoma [15]. In our case, the tumor demonstrated positive staining for S‐100, SOX‐10, Melan‐A, and HMB‐45.

Due to the rarity and heterogeneity of OMM, there is no standardized treatment protocol. Surgical excision is the mainstay for localized disease. However, advanced and metastatic cases require systemic therapy. Immune checkpoint inhibitors have shown promise in prolonging survival in patients with metastatic melanoma [16, 17]. Despite receiving combination immunotherapy, our patient experienced disease progression, prompting a transition to investigational therapy on a clinical trial and then palliative‐focused care.

Prognosis for oral melanoma remains poor, with reported 5‐year survival rates ranging from 15% to 38% [4]. In their 35‐case series, Lourenço et al. [18] similarly reported that most patients presented with advanced‐stage disease, contributing to unfavorable clinical outcomes. This is primarily due to its aggressive nature and early hematogenous and lymphatic spread to the lungs, liver, and regional lymph nodes [18, 19]. In this case, the patient′s disease was already advanced at initial presentation, with widespread metastases to the liver, lungs, spleen, and retroperitoneum, and later spread to the brain.

## 4. Conclusion

Oral melanoma is a rare form of cancer with poor prognosis and challenging to treat. Increased awareness for practitioners, especially general dentists, could improve detection, expedite care, and aid in better outcomes.

This case illustrates the impact of irregular dental checkups and patient′s lack of knowledge on timely diagnosis of a readily visible oral malignancy. Oral health care providers should recognize, appropriately assess, and accordingly monitor pigmented lesions of the oral cavity. Furthermore, this case highlights the importance of oral cavity inspection by medical providers, particularly when the primary source of visceral melanoma is unknown and detection of lesions in additional sites could potentially impact the therapeutic efficacy. Collaboration between medical and dental professionals could facilitate best case practices for meeting the needs of patients with oral melanoma.

**Nomenclature**
CT: computerized tomography
GERD: gastroesophageal reflux disease
OMM: oral mucosal melanoma
PET CT: positron emission tomography‐computed tomography

## Funding

No funding was received for this manuscript.

## Consent

Written informed consent was obtained from the patient for publication of this case report, as well as any accompanying clinical, radiographic, and histopathologic images.

## Conflicts of Interest

The authors declare no conflicts of interest.

## Data Availability

Data sharing not applicable to this article as no datasets were generated or analyzed during the current study.
